# Diphtheria and the So-called Anti-Toxine

**Published:** 1896-05

**Authors:** Joseph Fitz Mathew

**Affiliations:** Victoria, Pa.


### DIPHTHERIA AND THE SO-CALLED ANTI-TOXINE.

Jos. Fitz Mathew, M. D., Victoria, Pa.

There is every reason to believe that the obsequies of Behring's serum have commenced. The adverse testimony of many distinguished medical practitioners enjoying exceptional facilities for testing the Anti-toxine—whose mental faculties have resisted the influence of crack-brained bacteriological enthusiasts is such that it must now be regarded as little short of criminal to inject into the tissues this horse serum poisoned by diphtheritic toxine.

From various quarters we still hear favorable reports of its action, prematurely given, and it is unpleasant to reflect that the investment of $100 or less in an unfortunate horse will produce serum to the value of $2,000 to $2,500.

There was once a city in ancient times (beleaguered by an enemy, whose walls were out of repair, and the town council assembled to consult as to the best material to be used, when the currier, who had a large stock of leather on hand, said: "Gentlemen, in my opinion there is nothing like leather."—*Æsop's Fables*). Now let us hope that Behring's serum is not being recommended in some quarters from unworthy motives.

Assistant Surgeon F. O. B. Cordero, U. S. N., in reply to Surgeon-General of the U. S. N., said: "After a thorough study in various hospitals of Berlin of the use of Behring's diphtheria serum, so far proofs are lacking of the value of it in diphtheria. Children who during their first sickness have been treated with large doses of serum have, a short time afterward, acquired diphtheria anew. In a large number of cases children have been treated on the first and second day of their illness with the fullest doses of the Anti-toxine and died. * * * A large number of children treated with both large and small immunizing doses have within a few weeks acquired diphtheria, and some of them have died of it. * * * We do not possess a single scientific proof that a case of diphtheria was ever prevented by the immunizing process. It is certain that a large part of those who have died, notwithstanding the serum treatment, did not die from the effects of a mixed infection, but directly from the specific effects of the Klebs-Lœffler bacillus." Dr. Winters, of the Willard Parker Hospital, is most emphatic in his condemnation of it. He reports one hundred and fifty cases treated without the slightest effect. He further says: "The conditions of these babies was one of Anti-toxine scepticemia brought about by the influence of Anti-toxine on the blood."

A correspondent of the *New York Medical Record* gives a very unfavorable report from the hospitals of London. The doctor in charge of one of the largest of the seven hospitals devoted exclusively to infectious diseases, the Northwestern London Fever Hospital, said, that in common with other institutions, save one that is neutral, the Anti-toxine is now regarded as a complete failure, and on the whole rather harmful to patients. The mortality ranges as formerly, about twenty-seven per cent.

Dr. Lennox Brown, of London, reports the treatment of a hundred cases of diphtheria without Anti-toxine, and one hundred with it, with the same rate of mortality.

